# Considerations for Oral Cholera Vaccine Use during Outbreak after Earthquake in Haiti, 2010−2011

**DOI:** 10.3201/eid1711.110822

**Published:** 2011-11

**Authors:** Kashmira A. Date, Andrea Vicari, Terri B. Hyde, Eric Mintz, M. Carolina Danovaro-Holliday, Ariel Henry, Jordan W. Tappero, Thierry H. Roels, Joseph Abrams, Brenton T. Burkholder, Cuauhtémoc Ruiz-Matus, Jon Andrus, Vance Dietz

**Affiliations:** Centers for Disease Control and Prevention, Atlanta, Georgia, USA (K.A. Date, T.B. Hyde, E. Mintz, A. Henry, J.W. Tappero, T.H. Roels, J. Abrams, B.T. Burkholder, V. Dietz); Pan American Health Organization, Washington DC, USA (A. Vicari, M.C. Danovaro-Holliday, C. Ruiz-Matus, J. Andrus); Ministère de la Santé Publique et de la Population, Port-Au-Prince, Haiti (A. Henry)

**Keywords:** cholera, outbreak, cholera vaccination, oral cholera vaccine, OCV, bacteria, disaster, earthquake, waterborne infections, Haiti, vaccines, enteric infections

## Abstract

Oral cholera vaccines (OCVs) have been recommended in cholera-endemic settings and preemptively during outbreaks and complex emergencies. However, experience and guidelines for reactive use after an outbreak has started are limited. In 2010, after over a century without epidemic cholera, an outbreak was reported in Haiti after an earthquake. As intensive nonvaccine cholera control measures were initiated, the feasibility of OCV use was considered. We reviewed OCV characteristics and recommendations for their use and assessed global vaccine availability and capacity to implement a vaccination campaign. Real-time modeling was conducted to estimate vaccine impact. Ultimately, cholera vaccination was not implemented because of limited vaccine availability, complex logistical and operational challenges of a multidose regimen, and obstacles to conducting a campaign in a setting with population displacement and civil unrest. Use of OCVs is an option for cholera control; guidelines for their appropriate use in epidemic and emergency settings are urgently needed.

## Abstract 2

Many logistical and operational challenges prevented implementation of a vaccination campaign.

After an absence of over a century, cholera was reported in Haiti on October 22, 2010, in the Artibonite River valley (*1*). This happened within 9 months of the January 12 earthquake, which killed >222,000 persons and displaced an estimated 2 million around the capital city of Port-Au-Prince (*2*,*3*). Within 1 month, cholera was confirmed in all 10 Haitian departments, including spread to the earthquake-affected area (*1*,*4*).

Cholera is an acute, watery, diarrheal illness caused by the toxigenic bacterium *Vibrio cholerae* serogroups O1 and O139 and can be rapidly fatal if not promptly treated (*5*). Epidemic cholera is most often caused by fecally contaminated water (*5*). Disruptions in water and sanitation infrastructure after disasters (mainly flooding and cyclones) and overcrowding and precarious conditions caused by large population displacements may create an environment conducive to cholera’s rapid spread (*6*–*9*), although 1 report documents epidemic risk to be small after geophysical disasters (*10*). Proven measures for treatment (oral and intravenous rehydration and antimicrobial drugs in severe cases) and prevention (provision of safe water, community education, and improved access to sanitation and hygiene) are prioritized to reduce death and spread during the acute response to epidemic cholera (*11*,*12*). In the long term, increasing population coverage with improved drinking water sources and proper sanitation are the most effective means of preventing outbreaks of cholera and other enteric diseases (*5*).

Cholera vaccination is an additional key option for cholera prevention and control. In cholera-endemic countries, the targeted use of cholera vaccines is increasingly being recognized as a useful complement to improving water, sanitation, and hygiene (*13*). Guidelines for considering the use of cholera vaccines in complex humanitarian emergencies (*14*) and for their preemptive use to protect populations threatened by epidemic cholera have been proposed (*13*). Expert opinions differ on the applicability, feasibility, and impact of cholera reactive vaccination in epidemic situations (*14*–*19*); thus far, experience in these situations is limited to small outbreaks in stable populations (*20*,*21*). However, the value of cholera vaccines in controlling ongoing outbreaks through reactive vaccination is not yet established (*13*,*14*,*16*). Furthermore, vaccine use in outbreaks in post-disaster settings poses unique logistic, financial, and human resource challenges. Nonetheless, over the past decade, the occurrence of large, protracted outbreaks (*22*,*23*) and the licensing and marketing of new oral cholera vaccines (OCVs) have focused discussion on the role of vaccination as a supplementary cholera preventive and control measure (*13*). We describe the process used and the evidence reviewed by the US Centers for Disease Control and Prevention (CDC), the Pan American Health Organization (PAHO), and the Haitian Ministère de la Santé Publique et de la Population (MSPP) (Haitian Ministry of Public Health and Population), when considering OCV use during the 2010–2011 cholera outbreak in Haiti.

## The Study

### Decision-making Process and Development of Recommendations for OCV Use in Haiti, October–December 2010

Immediately after notification of the outbreak, an emergency response was launched by MSPP with assistance from CDC, PAHO, and other governmental and nongovernmental organizations; initial discussions regarding the potential role and use of OCVs occurred within days. In subsequent weeks, PAHO and CDC convened working groups and expert advisory committees to review vaccine characteristics, World Health Organization (WHO) position papers and recommendations, published experience with OCV use in complex emergency settings, global vaccine availability, and logistical implications. The most current information regarding vaccine availability was sought from vaccine manufacturers and other partners, and the latest assessments of Haiti’s postearthquake vaccine deployment capacity were obtained from agencies working in Haiti. Initial recommendations, presented to MSPP on October 27, 2010, and November 1, 2010, by PAHO and CDC, respectively, did not support cholera vaccination because of pressing needs for priority interventions of safe water provision and cholera treatment measures, and limited immediate vaccine availability (Technical Appendix 1).

In mid-December 2010, the initial recommendations were revisited for several reasons: 1) clinical training and priority interventions for treatment and improved water quality had been established; 2) rolling 14-day hospital case-fatality rates had decreased from 4% in early November to ≈1.5% by mid-December, suggesting improved access to treatment; 3) relatively few cases were reported from Port-au-Prince, including those in internally displaced persons camps, suggesting that a large population remained at risk; and 4) anecdotal information indicated that additional vaccine supply might soon become available. On December 17, 2010, PAHO convened an ad hoc consultation with international experts and other key stakeholders to reconsider options for OCV use in Haiti, given the situation at that time (*24*).

Real-time modeling was conducted by CDC during the course of the outbreak to develop preliminary estimates of numbers of cases and hospitalizations for planning purposes. Early in the outbreak, an epidemic model fit to the first 7 weeks of cholera surveillance data was created to develop preliminary OCV impact estimations, the details of which are described in Technical Appendix 2.

### OCV Characteristics and Status

The 2 available OCVs, Dukoral (Crucell, Stockholm, Sweden), and Shanchol (Shantha Biotechnics, Hyderabad, India) are whole-cell, killed vaccines. Key vaccine characteristics are summarized in Table 1. Both vaccines require 2 doses (3 doses of Dukoral are required for children 2–5 years of age) administered about 7–14 days apart (up to 42 days apart for Dukoral). Dukoral doses must be administered with buffer that requires 75–150 mL of clean water; Shanchol does not require buffer. Both vaccines require cold chain maintenance and have packed volumes larger than those of other Expanded Program on Immunization (EPI) vaccines (estimated for Dukoral to be 30× larger than those of the EPI vaccines), indicating the need for greater cold chain capacity (*13*) (see Technical Appendix 1 for additional references).

**Table 1:** Salient features of oral cholera vaccines available as of December 31, 2010*

Feature	Dukoral†	Shanchol
Composition	Monovalent formalin-based heat-killed whole cells of *Vibrio cholerae* O1 (classical and El Tor, Inaba and Ogawa) + recombinant cholera toxin B subunit	Bivalent, killed whole cells of *V. cholerae* O1 (Inaba and Ogawa, classical and El Tor) and O139
Number of doses for full immunization	2 doses (3 doses in children 2–5 y)	2 doses
Schedule	7–14 d apart (up to 42 d apart)	14 d apart
Age for vaccination per licensure	>2 y	>1 y
Administration	Oral with buffer	Oral
Requirement for buffer and water	Yes (adults,150 mL; children 2–5 y, 75 mL)	No (water may be used)
Food and water restrictions before and after vaccination	No food or water 1 h before and after ingestion of vaccine	None
Packaging	3-mL single dose vials (vaccine) + effervescent granules in a sachet (buffer)	1.5-mL single dose vial
Cold-chain and other storage requirements	2–8°C; additional storage space for water (not in cold chain)	2–8°C
Shelf life	3 y at 2–8°C; stable for 1 mo at 37°C	2 y at 2–8°C
WHO prequalified	Yes	No
Cost of vaccine	US $6 per dose ($12–$18 for full series, i.e., for 2–3 doses); price quoted for Haiti in January 2011, $3.64 per dose	$1.85 per dose ($3.70 for full series)
Safety profile	High	High
Earliest onset of protection	7–10 d after full immunization	7–10 d after full immunization per manufacturer
Efficacy and effectiveness	Matlab trial, Bangladesh: 85% at 4–6 mo; 62% at 1 y, 58% at 2 y, 18% at 3 y; in 2–5 y olds: 100% at 4–6 mo, 38% at 1 y; military center, Peru: 86% at 4–5 mo; outskirts of Lima, Peru: 60% at 2 y; Beira, Mozambique: 85% with 2 doses, 78% with >1 dose at 1–6 mo	Kolkata, India: 67% at 2-y follow-up with 2 doses
Single dose effectiveness	Low; Matlab trial, Bangladesh: 12% during 3 y (lower limit of 95% confidence interval –29%)	Unknown studies planned
Herd protection	Yes	Expected but not yet demonstrated

*Other oral cholera vaccines not summarized: An injectable vaccine may be available in some countries, but is not recommended by the World Health Organization (WHO) because of its reactogenicity, limited efficacy, and short duration of protection; mORCVAX, similar to Shanchol, is licensed in Vietnam but is not eligible for WHO prequalification, which restricts its global utilization; a single-dose, oral, live attenuated cholera vaccine (CVD 103-HgR: Orochol, Mutachol) by Crucell/Berna Biotech is no longer manufactured. Several new oral cholera vaccines, intended to be administered as a single dose, are in different stages of development and licensure. However, these vaccines in the most advanced stages of development, including Peru-15 (USA and China), *V. cholerae* 638 (Cuba), and VA1.4 (India), are at least a few years away from becoming widely available. †Includes data from early vaccine trials of whole-cell recombinant beta subunit and whole-cell beta subunit vaccine for Dukoral.

At the time, only Dukoral was prequalified by WHO and since 1991 has been licensed in ≈60 countries for persons >2 years old. The newer Shanchol vaccine, licensed in India since 2009 for persons >1 year of age, was pending WHO prequalification. WHO prequalification is required for vaccine procurement by United Nations agencies, including the PAHO Revolving Fund, the United Nations Children’s Fund, and for some donor funding, including the US government.

Both OCVs have been shown to be safe and immunogenic; clinical trials demonstrated protective efficacy of 66%–85% after 2 doses but almost none after a single dose. Protection is achieved ≈7 days following the last dose of Dukoral (estimated to be similar for Shanchol) and persists for ≈2 years. Herd protection has been inferred for Dukoral according to a reanalysis of the Bangladesh original clinical trial data and has been suggested to be substantial in cholera-endemic areas. A similar herd-protection effect with Shanchol, although expected, has not yet been studied. Most vaccine effectiveness studies have been conducted in cholera-endemic settings, where some level of preexisting population immunity can be expected because of recurrent exposure. These study results are in contrast to Haiti, where the population was immunologically naive to cholera until the current epidemic, suggesting a need for higher vaccination coverage with the full series to achieve the suggested herd protection. One study conducted among Peruvian military recruits (an immunologically naive population similar to that in Haiti) shows promise; the vaccine demonstrated an 86% protective efficacy at 4–5 months. Studies have shown these vaccines to be cost-effective in cholera-endemic areas only when herd-protection effects are considered.

Immunity after natural cholera infection is incomplete and, particularly after a first infection and after infection with the El Tor biotype, appears to be of relatively short duration, waning within 6 years in contrast to life-long immunity conferred by viral infections. Nevertheless, the duration of protection with natural infection is longer than that conferred by OCVs.

### Global OCV Availability

During the initial weeks of the outbreak, an estimated 100,000–300,000 doses of Dukoral and 150,000 doses of Shanchol were available for immediate shipment, and an estimated 1,000,000 additional doses could have been made available over a 1-year period (International Vaccine Institute, Crucell, PAHO, pers. comm.). In December 2010 and January 2011, both manufacturers indicated that a larger supply (up to 5 million combined doses for both vaccines) (*25*) could be made available gradually over 1–3 years, but firm orders and financial commitments were needed before production capacity could increase.

### Previous Experiences with Mass OCV Campaigns in Complex Emergency Settings

Mass OCV campaigns have been conducted in complex emergency settings, with mixed results (*14**,**16*; Technical Appendix 2) Two such experiences, in Sudan and Indonesia, formed the basis for the 2005 WHO recommendations for cholera vaccine use in complex emergency situations (Table 2). Both were preemptive campaigns; however, the effectiveness of the intervention was not evaluated in either setting.

**Table 2:** WHO recommendations for cholera vaccination in complex emergencies, 2005*

• The relevance of cholera vaccination should be examined in light of other public health priorities. If vaccination is deemed necessary, water and sanitation programs should be implemented before or concurrently with vaccination.
• A high level commitment by all stakeholders and national authorities is critical.
• Vaccination with the current prequalified vaccine is not recommended by WHO once an outbreak has started, because of logistic and operational challenges.
• Vaccination campaign should not interfere with other critical public health interventions.
• Other exclusions for vaccination would include these criteria: high mortality from other causes; basic unmet needs of water, food and shelter; an ongoing outbreak of other disease; untenable security situation.

*WHO, World Health Organization.

In 2004, a small-scale mass vaccination campaign in Darfur, Sudan, that focused on 55,000 persons in well-organized refugee camps with limited population movements was deemed feasible because there was strong political and partner commitment, easy access to the intended population, and widespread community mobilization. The campaign lasted ≈2 months, achieved 87% 2-dose vaccination coverage, and cost US$7.10 per fully immunized person, including $6.40 for vaccine purchase and delivery and $0.70 for indirect campaign costs. A prior (1997) OCV campaign targeting 44,000 Sudanese refugees in a similar stable refugee setting in Uganda had also demonstrated feasibility, low indirect campaign costs ($0.53), and high coverage.

In 2005 post-tsunami Aceh, Indonesia, a preemptive vaccination campaign for ≈79,000 persons lasted 6 months, achieved 2-dose coverage of 69%, and cost US$18 per fully immunized person, with >$8.15 being indirect campaign costs. Here, in addition to large cold-chain volume requirements, the need for clean water for administration with the vaccine, 12% vaccine wastage, and difficulty reaching persons for the second dose, other obstacles included infrastructure destruction, disaster-related loss of critical human resources, and high population movements, conditions similar to those in Haiti in 2010 when the cholera outbreak began.

### Current WHO Position on OCV Use

In March 2010, WHO issued a revised position statement regarding OCV use in disease-endemic and outbreak settings (*13*). WHO recommends OCV use in endemic settings, in conjunction with other prevention and control strategies, but the organization’s position on OCV use in epidemic cholera settings is less conclusive. In outbreak situations and during complex emergencies, WHO states that pre-emptive vaccination, in areas determined to be at imminent risk for infection, should be considered after taking into account the local epidemiologic context and capacity to mount a vaccination campaign. However, given the limited experience, WHO states that reactive vaccination could be considered in affected areas. To guide health authorities regarding OCV use during complex emergencies, WHO proposes a 3-step predictive risk assessment approach, which considers 1) the risk for cholera outbreak, 2) outbreak containment capacity, and 3) the feasibility of conducting a mass vaccination campaign (*14*).

### Situation and Vaccine Deployment Capacity in Haiti

Haiti is the third largest and third most populous country in the Caribbean, with a population of ≈10 million persons living in 10 administrative departments, and has long been the poorest country in the Americas with remarkably low socioeconomic and health indicators compared with the rest of the region (*26*). Poor access to basic health care services has been evident from recently reported 2009 routine EPI (http://www.who.int/immunization_delivery/en) coverage of 68% for third dose of diptheria-pertussis-tetanus vaccine and 60% for the first dose of measles-containing vaccine (*27*). In 2008, only 63% of the Haitian population had access to improved water sources (such as a protected well or piped water) with only 12% receiving treated, piped water and only 17% having access to adequate sanitation (*28*). Diarrhea has been the leading cause of death among Haitian children <5 years of age (*29*), and, given the limited access to clean water and sanitation, rapid, sustained cholera transmission after introduction of the disease is not surprising (*30*). Postearthquake loss of infrastructure and human capacity, coupled with massive population displacement, worsened these preexisting conditions.

The enormous destruction caused by the earthquake disrupted the ability to initiate large-scale interventions in Haiti. A postearthquake immunization campaign using measles–rubella and diphtheria–tetanus toxoid vaccines to address the immediate threat of vaccine-preventable diseases encountered challenges that included difficulty reaching a large target population, time needed for completion (≈4 months), and achieving adequate coverage (*31*), problems that could also hamper a cholera vaccine campaign.

**Figure 1:**
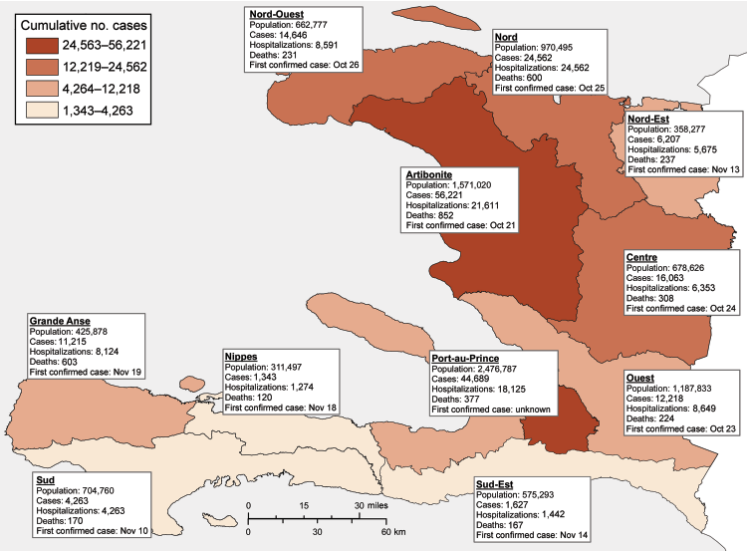
Distribution of cases of cholera among departments in Haiti, October 2010–January 16, 2011. Department population, earliest known date of confirmed case, and number of hospitalizations and deaths are indicated. Totals for Haiti: population, 9,923,243; cholera cases, 194,095; hospitalizations, 109,015; deaths: 3,889. Port-au-Prince includes the following communes: Carrefour, Cité Soleil, Delmas, Kenscoff, Petion-Ville, Port-au-Prince, and Tabarre. Data sources: Ministère de la Santé Publique et de la Population, Institut Haitien de Statistique et d’Informatique, Centre National de l’Information Géo Spatiale, and Laboratoire National De Santé Publique.

**Figure 2:**
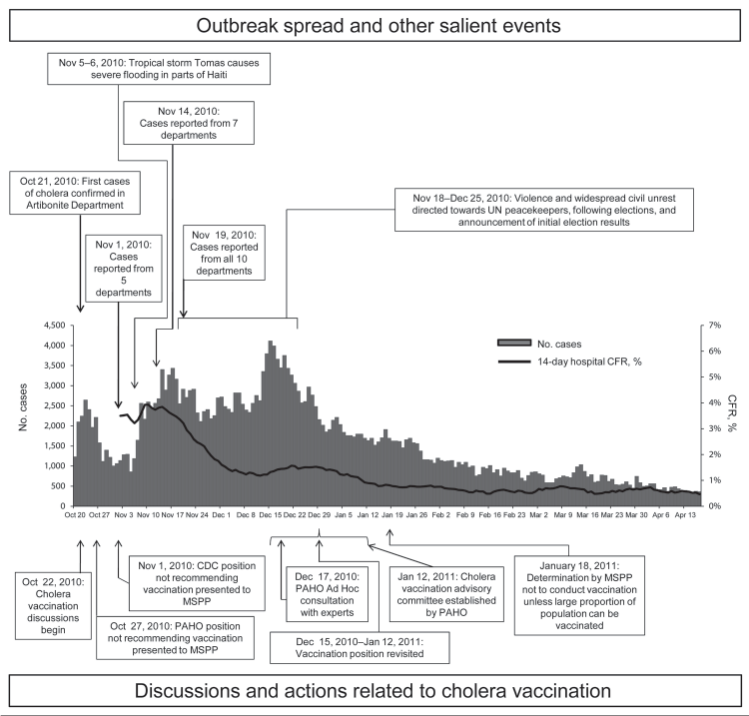
Events and actions related to considerations for cholera vaccination, Haiti, October 2010–April 2011. The full epicurve after January 18 is shown for reference only. Events and discussions regarding vaccination or other events after that date are not depicted. UN, United Nations; CFR, case-fatality rate; CDC, Centers for Disease Control and Prevention; MSPP, Haiti Ministère de Santé Publique et de la Population; PAHO, Pan American Health Organization.

An assessment of vaccine deployment capacity indicated lack of adequate cold-chain capacity and a critical shortage of human resources. Furthermore, identification of a well-defined target population, a major prerequisite for OCV campaigns (*14*), was difficult because the outbreak had spread rapidly to all 10 departments within ≈1 month (*4*); >1,000 cases were confirmed in each department by January 16, 2011 (Figure 1). Age-group and gender-specific attack rates suggested that both sexes and all age groups were at similar risk (*4*). Complicating the post-disaster situation, Tropical Storm Tomas caused severe flooding in parts of the country on November 5–6, 2010 (*32*). In addition, violence and widespread civil unrest directed toward United Nations peacekeepers, who were perceived as having introduced cholera to Haiti, occurred on November 18 (*33*). Additional protests erupted before the presidential election on November 28 and after announcements of the initial results on December 8, disrupting communications and travel for several days (*34*). The timeline of the cholera epidemic, with salient outbreak, meteorologic, social, and political events, and vaccination-related events is depicted in Figure 2. Results of the CDC Preliminary Real Time Modeling program for Projected Vaccine Impact are included in Technical Appendix 2.

### Cholera Vaccination Recommendations for Haiti

During the early phase of the outbreak, CDC and PAHO did not recommend cholera vaccination because of the severe challenges in preventing death among case-patients and controlling the rapid spread of the epidemic, constraints on available vaccine supply and on vaccine delivery resources, and the time needed from vaccine administration to development of protective immunity. In mid-December, when deaths among case-patients had decreased and vaccination recommendations were revisited, complex logistical and operational considerations and ongoing vaccine supply limitations led to recommendations to consider pilot intervention studies by some groups. On January 18, 2011, MSPP decided that cholera vaccination would be considered for Haiti only if sufficient vaccine (>1 million doses) were available to immunize a large proportion of the population with a goal to eventually reach 6 million persons (*35*; Technical Appendix 1).

## Discussion and Conclusions

Our effort highlights the in-depth consideration given to the possibility of using cholera vaccination for controlling the outbreak in Haiti, which was also considered multiple times by many partners. To date, although small-scale demonstration projects have been proposed and larger scale campaigns have been called for, cholera vaccine has not yet been used in Haiti.

Numerous challenges were identified, and efforts were made to assess them. Obstacles to vaccine use included limited resources to address the acute need for medical treatment and basic prevention services during the early epidemic phase, a limited supply of available vaccine and of WHO-prequalified vaccine, the complex planning and logistics that the 2-dose vaccine requires, and political opposition to anything less than a nationwide immunization campaign. Furthermore, identification of an equitable and politically acceptable target population for this limited vaccine supply was not possible in the heat of the epidemic, resulting in MSPP’s rejection of proposals for small-scale demonstration projects.

The logistics of organizing a multidose vaccination campaign in a setting characterized by shortages of human resources, cold-chain capacity, and health system infrastructure and by a large, displaced, highly mobile population were also limitations. Additional vaccine-related issues included the need for clean water for Dukoral administration and the relatively long interval after vaccination before immunity develops. A single-dose vaccine that can be administered without water would be much easier to deliver. Promising data from a Shanchol immunogenicity study in cholera-endemic Kolkata, India, found that vibriocidal antibody responses after 1 dose were equivalent to those seen after 2 doses (*36*); however, actual efficacy trials in populations previously unexposed to cholera are needed before 1dose of Shanchol could be considered for epidemic control in Haiti.

Lack of WHO prequalification was an additional impediment to use of the Shanchol vaccine in Haiti. Haiti procures vaccines through the United Nations Children’s Fund, which only purchases WHO-prequalified vaccines. A decision to use a nonprequalified vaccine would require direct vaccine procurement by the Haitian government or donation by manufacturers or donors. In December 2009, the WHO Strategic Advisory Group of Experts on Immunization recommended that Shanchol be prioritized for prequalification (Technical Appendix 1). Accelerated prequalification of cholera vaccines will be helpful for large-scale manufacturing and will reduce delays in obtaining vaccine for wider global use.

A well-defined public communications strategy in advance of a vaccine campaign would have been helpful in Haiti because the local population had no previous experience with cholera, and it was widely believed to have been introduced by external aid agencies. Active and timely monitoring of and to Adverse Events Following Immunization during a campaign would be essential; however, instituting such a monitoring system would have been problematic because of the volatile political situation with widespread unrest, which created insecurities with field operations.

Preliminary CDC real-time modeling estimates in December 2010, using data from the first 7 weeks of the outbreak, predicted only a marginal impact for outbreak control with the immediately available vaccine supply. This model had several limitations (described in detail in Technical Appendix 2). It was a real-time effort conducted during the early response phase for planning, resource allocation, and preliminary decision making, when sparse outbreak data were available, according to conservative assumptions, which may have underestimated the impact of vaccination. In contrast, other recently published disease models using additional outbreak data and different assumptions suggest that substantial health gains could be achieved by reactive cholera vaccination. Although promising, these models may not be fully applicable to the Haitian situation: some use data from cholera-endemic countries or assumptions that may not have been consistent with the situation in Haiti. Modeling may be useful for identifying appropriate indications for reactive OCV use in the future, particularly if precise and detailed surveillance data, which accurately reflect field conditions, are available for modeling early in an epidemic.

The careful consideration of cholera vaccination for outbreak control in Haiti yielded valuable lessons. For example, inadequate stocks of prequalified cholera vaccine prompted discussion of the establishment of a global cholera vaccine stockpile (Technical Appendix 2) to help reduce the projected high costs of mass vaccination and overcome the inability of manufacturers to produce large stocks without a firm demand. Earlier considerations regarding the utility and relevance of a cholera vaccine stockpile indicated the need for precise guidelines for its establishment and management, accurate vaccine demand projections, and cost-effectiveness estimates (Technical Appendix 2). The issue of equitable vaccine distribution of available global OCV supplies is essential, especially in the context of simultaneous multinational outbreaks and vaccine demands. For example, cholera outbreaks were reported in several countries coincident with the outbreak in Haiti (Technical Appendix 2). Creating a stockpile for cholera vaccines will, therefore, require engagement of the broader global community and development of practical guidelines and strategies for its use.

WHO guidelines on control of cholera outbreaks note a potential role for pre-emptive and reactive vaccination as part of comprehensive public health intervention measures (*13*). However, as the situation in Haiti demonstrates, additional guidelines are needed on the relative priority for cholera vaccine use as outbreaks rapidly evolve in a variety of epidemiologic situations. The 3-step decision-making tool originally developed in 2005 (*14*) provides general guidance for decision making on the use of cholera vaccine before an outbreak but was not easily applicable to the complexities of the specific situation in Haiti. Modeling and field experience can help inform revisions of the WHO decision-making tool.

OCVs remain an option for cholera control globally and in Haiti, where OCVs could potentially be used to dampen the recurrence of cholera in the years to come. However, cholera vaccination should be considered in the context of the introduction of other new and underutilized vaccines and must take into account the potentially competing resource needs of the routine national immunization program. If cholera becomes endemic to Haiti, the projected preventable disease prevalence and cost-effectiveness are critical issues that will inform cholera vaccine introduction (*37*). The Strategic Advisory Group of Experts on Immunization and the Global Alliance for Vaccines and Immunization have recommended the preparation of an investment case for potential donors and national and international organizations, to provide critical information regarding OCVs and to highlight potential demand and funding gaps (*38*,*39*).

Cholera vaccination must be synergistic with other cholera prevention and control measures, and studies are ongoing to evaluate this effect (*40*). But these cohesive efforts are challenging in the context of rapidly expanding epidemics in complex post-disaster situations, where resources for essential surveillance, treatment, and other nonvaccine control measures quickly become depleted. Reactive OCV use in the setting of an outbreak requires consideration of multiple issues unique to each situation. The feasibility of OCV use has been demonstrated in other stable refugee settings (*16*) and 1 small-scale outbreak setting (*21*). Successful efforts by national and international agencies to introduce and expand the use of cholera vaccines in outbreak and post-disaster settings will depend on clear, well-informed, and specific guidelines to help countries and donors make appropriate decisions regarding reactive OCV use.

## Supplementary Material

Technical Appendix 1 Additional World Health Organization (WHO) Documents.

Technical Appendix 2 Real-Time Modeling of Estimated Cholera Vaccine Impact on the Cholera Outbreak in Haiti, Centers for Disease Control and Prevention, December 2010.

## References

[1] Centers for Disease Control and Prevention. Update: cholera outbreak—Haiti, 2010. MMWR Morb Mortal Wkly Rep. 2010;59:1473–9. 21085088

[2] Inter-Agency Standing Committee. Response to the humanitarian crisis in Haiti following the 12 January 2010 earthquake. 2010 [cited 2011 Sep 22]. http://www.humanitarianinfo.org/iasc/pageloader.aspx?page=content-news-newsdetails&newsid=143

[3] Centers for Disease Control and Prevention. Rapid establishment of an internally displaced persons disease surveillance system after an earthquake—Haiti, 2010. MMWR Morb Mortal Wkly Rep. 2010;59:939–45. 20689498

[4] Centers for Disease Control and Prevention. Update: outbreak of cholera—Haiti, 2010. MMWR Morb Mortal Wkly Rep. 2010;59:1586–90. 21150867

[5] Menon M, Mintz E, Tauxe R. Cholera. In: Brachman P, Abrutyn E, editors. Bacterial infections of humans; 4th ed. New York: Springer Science; 2009. p. 249–72.

[6] Bompangue D, Giraudoux P, Piarroux M, Mutombo G, Shamavu R, Sudre B, Cholera epidemics, war and disasters around Goma and Lake Kivu: an eight-year survey. PLoS Negl Trop Dis. 2009;3:e436. 10.1371/journal.pntd.0000436 19436726 PMC2677153

[7] Schwartz BS, Harris JB, Khan AI, Larocque RC, Sack DA, Malek MA, Diarrheal epidemics in Dhaka, Bangladesh during three consecutive floods: 1988, 1998, and 2004. Am J Trop Med Hyg. 2006;74:1067–73. 16760521 PMC1626162

[8] Watson JT. Connolly. Epidemics after natural disasters. Emerg Infect Dis. 2007;13:1–5. 10.3201/eid1301.060779 17370508 PMC2725828

[9] Panda S, Pati KK, Bhattacharya MK, Koley H, Pahari S, Nair GB. Rapid situation & response assessment of diarrhoea outbreak in a coastal district following tropical cyclone AILA in India. Indian J Med Res. 2011;133:395–400. 21537092 PMC3103172

[10] Floret N, Viel JF, Mauny F, Hoen B, Piarroux R. Negligible risk for epidemics after geophysical disasters. Emerg Infect Dis. 2006;12:543–8. 16704799 10.3201/eid1204.051569 PMC3294713

[11] World Health Organization. Global Task Force on Cholera Control. First steps for managing an outbreak of acute diarrhoea. 2004 [cited 2010 Nov 24]. http://www.who.int/topics/cholera/publications/en/first_steps.pdf

[12] World Health Organization. Global Taskforce on Cholera Control. Cholera outbreak: assessing the outbreak response and improving preparedness. 2004 [cited 2010 Nov 29]. http://whqlibdoc.who.int/hq/2004/WHO_CDS_CPE_ZFk_2004.4_eng.pdf

[13] World Health Organization. Cholera vaccines: WHO position paper. Geneva: The Organization; 2010 [2010 Mar 26]. http://www.who.int/wer/2010/wer8513.pdf

[14] World Health Organization. Oral cholera vaccine use in complex emergencies: what next? Report of WHO meeting, 14–16 Dec 2005. Cairo (Egypt): The Organization; 2006.

[15] Chaignat C-L. What about cholera vaccines? Expert Rev Vaccines. 2008;7:403–5. 10.1586/14760584.7.4.403 18444886

[16] Chaignat C-L, Monti V, Soepardi J, Petersen G, Sorensen E, Narain J, Cholera in disasters: do vaccines prompt new hopes? Expert Rev Vaccines. 2008;7:431–5. 10.1586/14760584.7.4.431 18444890

[17] Clemens J, Holmgren J. Urgent need of cholera vaccines in public health-control programs. Future Microbiol. 2009;4:381–5. 10.2217/fmb.09.9 19416006

[18] Enserink M. Public health: no vaccines in the time of cholera. Science. 2010;329:1462–3. 10.1126/science.329.5998.1462 20847246

[19] Bhattacharya S, Black R, Bourgeois L, Clemens J, Cravioto A, Deen JL, The cholera crisis in Africa. Science. 2009;324:885. 10.1126/science.1173890 19443768

[20] Calain P, Chaine J-P, Johnson E, Hawley M-L, O’Leary MJ, Oshitani H, Can oral cholera vaccination play a role in controlling a cholera outbreak? Vaccine. 2004;22:2444–51. 10.1016/j.vaccine.2003.11.070 15193408

[21] Anh DD, Lopez AL, Thiem VD, Grahek SL, Duong TN, Park JK, Use of oral cholera vaccines in an outbreak in Vietnam: a case control study. PLoS Negl Trop Dis. 2011;5:e1006. 10.1371/journal.pntd.0001006 21283616 PMC3026769

[22] World Health Organization. Cholera annual report 2009. WHO weekly epidemiological record. Geneva: The Organization; 2010.

[23] World Health Organization. Cholera annual report 2009. WHO weekly epidemiological record. The Organization: WHO; 2007.

[24] Danovaro-Holliday MC. Ad-hoc scientific consultation on potential role of cholera vaccination in the Americas in the context of the 2010 outbreak in the Hispaniola island. Global Immunization News. Geneva: World Health Organization; 2011.

[25] Ruiz Matus C. Challenges of use of cholera vaccines in Haiti and the Americas. 2011 [cited 2011 Sep 22]. http://www.who.int/immunization/sage/SAGE_April_2011_cholera_haiti_paho.pdf

[26] Pan American Health Organization. Haiti: population health assessment prior to the 2010 earthquake; Jan 2010. Washington: The Organization; 2010.

[27] Pan American Health Organization. Immunization in the Americas: 2010 summary. Washington: The Organization; 2010.

[28] World Health Organization. UNICEF. Progress on sanitation and drinking water: 2010 update. Geneva: The Organization; 2010.

[29] World Health Organization. Haiti: health profile: Geneva: The Organization; 2008.

[30] Ackers M-L, Quick RE, Drasbek CJ, Hutwagner L, Tauxe RV. Are there national risk factors for epidemic cholera? The correlation between socioeconomic and demographic indices and cholera incidence in Latin America. Int J Epidemiol. 1998;27:330–4. 10.1093/ije/27.2.330 9602419

[31] Pan American Health Organization. Immunization Newsletter. 2010 Aug [cited 2011 Sep 22] http://new.paho.org/hq/dmdocuments/2011/SNE3204.pdf

[32] Kusher J. Hurricane Tomas floods quake shattered town. Sign On San Diego. 2010 Nov 5 [cited 2011 Apr 20]. http://www.signonsandiego.com/news/2010/nov/05/hurricane-tomas-floods-quake-shattered-haiti-town/

[33] Gutman M. Haiti: As cholera spreads, frustration builds. ABCNewscom. 2010 Nov 18 [cited 2011 Sep 22]. http://abcnews.go.com/International/haiti-cholera-epidemic-sparks-anger-civil-unrest/story?id=12185574

[34] Crane J. Post-election violence spreads across the country. 2010 Dec 9 [cited 2011 Sep 22]. http://www.france24.com/en/20101208-haiti-post-election-violence-spreads-port-au-prince-manigat-celestin-martelly

[35] Cyranoski D. Cholera vaccine plan splits experts. Nature. 2011;469:273–4. 10.1038/469273a 21248807

[36] Kanungo S, Paisley A, Lopez AL, Bhattacharya M, Manna B, Kim DR, Immune responses following one and two doses of the reformulated, bivalent, killed, whole-cell, oral cholera vaccine among adults and children in Kolkata, India: a randomized, placebo-controlled trial. Vaccine. 2009;27:6887–93. 10.1016/j.vaccine.2009.09.008 19761838

[37] World Health Organization. Vaccine Introduction guidelines. Adding a vaccine to a national immunization programme: decision and implementation. Geneva: The Organization; November 2005.

[38] World Health Organization. Meeting of the Strategic Advisory Group of Experts on Immunization, October 2009—conclusions and recommendations [cited 2011 Sep 22]. http://www.who.int/wer/2009/wer8450.pdf.

[39] Applied Strategies Consulting. GAVI Vaccine Investment Strategy: Cholera analysis: Final: October 27, 2008 GAVI. 2008.

[40] Marshall A. Bangladesh, a new way to fight cholera. 2011 [cited 2010 Aug 10]. http://www.time.com/time/world/article/0,8599,2048937,00.html

