# Supplementary material for: Considerations for Oral Cholera Vaccine Use during Outbreak after Earthquake in Haiti, 2010−2011
Source: Emerg Infect Dis. 2011 Nov;17(11):2105–12. doi: 10.3201/eid1711.110822 (PMC3310586; doi:10.3201/eid1711.110822)
Supplement: Technical Appendix 1 — Additional World Health Organization (WHO) Documents. [file 11-0822-Techapp1_4p.pdf]

Article DOI: http://dx.doi.org/10.3201/eid1711.11082211

Considerations for Oral Cholera Vaccine Use during 2010–2011 Outbreak after Earthquake in Haiti

Technical Appendix 1

Bibliography

Additional World Health Organization (WHO) Documents

World Health Organization. Meeting of the Strategic Advisory Group of Experts on Immunization, October 2009—conclusions and recommendations. Geneva: World Health Organization; 11 December 2009.

World Health Organization. Potential use of oral cholera vaccines in emergency situations. Report of a WHO meeting. Geneva: World Health Organization; May 1999.

PAHO and CDC Position Recommendations for Oral Cholera Vaccine Use in Haiti Early During the Outbreak in 2010

Pan American Health Organization. PAHO Position on Cholera Vaccination in Haiti, Version October 27, 2010: Pan American Health Organization; 2010.

Centers for Disease Control and Prevention. CDC Memo to the Record: Cholera vaccination in the context of the outbreak in Haiti (unpublished). November 1, 2010.

Additional Discussions and Ministère de la Santé Publique et de la Population (MSPP) Position on Cholera Vaccine Use for Haiti

Danovaro-Holliday MC. Ad-hoc scientific consultation on potential role of cholera vaccination in the Americas in the context of the 2010 outbreak in the Hispaniola island. Global Immunization News. Geneva: World Health Organization (WHO). 28 January 2011.

Pan American Health Organization. Experts Call for International Cholera Vaccine Stockpile. 2010 [cited April 20, 2011]. http://new.paho.org/hq/index2.php?option=com_content&do_pdf=1&id=4569

Cyranoski D. Cholera vaccine plan splits experts. Nature. 2011;469:273–4. PubMed doi:10.1038/469273a

Oral Cholera Vaccines Characteristics and Status

Shanchol Product Information. 2009 [cited 2010 Nov 22]. http://www.shanthabiotech.com/images/Shanchol%20PI%202of2.jpg

World Health Organization. Use of the two-dose oral cholera vaccine in the context of a major natural disaster. Report of a mass vaccination campaign in Aceh Province, Indonesia, 2005. Geneva: World Health Organization; 2006.

Clemens JD, Sack DA, Harris JR, van Loon F, Chakraborty J, Ahmed F, et al. Field trial of oral cholera vaccines in Bangladesh: results from three-year follow-up. Lancet. 1990;335:270–3. PubMed doi:10.1016/0140-6736(90)90080-O

Lucas MES, Deen JL, von Seidlein L, Wang X-Y, Ampuero J, Puri M, et al. Effectiveness of Mass Oral Cholera Vaccination in Beira, Mozambique. N Engl J Med. 2005;352:757–67. PubMed doi:10.1056/NEJMoa043323

Sur D, Lopez AL, Kanungo S, Paisley A, Manna B, Ali M, et al. Efficacy and safety of a modified killed-whole-cell oral cholera vaccine in India: an interim analysis of a cluster-randomised, double-blind, placebo-controlled trial. Lancet. 2009;374:1694–702. PubMed doi:10.1016/S0140-6736(09)61297-6

van Loon FPL, Clemens JD, Chakraborty J, Rao MR, Kay BA, Sack DA, et al. Field trial of inactivated oral cholera vaccines in Bangladesh: results from 5 years of follow-up. Vaccine. 1996;14:162–6. PubMed doi:10.1016/0264-410X(95)00122-H

Sanchez JL, Vasquez B, Begue RE, Meza R, Castellares G, Cabezas C, et al. Protective efficacy of oral whole-cell/recombinant-B-subunit cholera vaccine in Peruvian military recruits. Lancet. 1994;344:1273–6. PubMed doi:10.1016/S0140-6736(94)90755-2

Taylor DN, Cárdenas V, Sanchez JL, Bégué RE, Gilman R, Bautista C, et al. Two-year study of the protective efficacy of the oral whole cell plus recombinant B subunit cholera vaccine in Peru. J Infect Dis. 2000;181:1667–73. PubMed doi:10.1086/315462

Ali M, Emch M, von Seidlein L, Yunus M, Sack DA, Rao M, et al. Herd immunity conferred by killed oral cholera vaccines in Bangladesh: a reanalysis. Lancet. 2005;366:44–9. PubMed doi:10.1016/S0140-6736(05)66550-6

Longini IM Jr, Nizam A, Ali M, Yunus M, Shenvi N, Clemens JD. Controlling endemic cholera with oral vaccines. PLoS Med. 2007;4:e336. PubMed doi:10.1371/journal.pmed.0040336

Jeuland M, Cook J, Poulos C, Clemens J, Whittington D, Group DCES. Cost-effectiveness of new-generation oral cholera vaccines: a multisite analysis. Value Health. 2009;12:899–908. PubMed doi:10.1111/j.1524-4733.2009.00562.x

Prequalification of Vaccines for United Nations Purchase

World Health Organization. A system for the prequalification of vaccines for UN supply. 2010 [cited December 1, 2010]; http://www.who.int/immunization_standards/vaccine_quality/pq_system/en/index.html

DeRoeck D, Bawazir SA, Carrasco P, Kaddar M, Brooks A, Fitzsimmons J, et al. Regional group purchasing of vaccines: review of the Pan American Health Organization EPI revolving fund and the Gulf Cooperation Council group purchasing program. Int J Health Plann Manage. 2006;21:23–43. PubMed doi:10.1002/hpm.822

Immunity With Natural Cholera Infection

Clemens JD, van Loon F, Sack DA, Rao MR, Ahmed F, Chakraborty J, et al. Biotype as determinant of natural immunising effect of cholera. Lancet. 1991;337:883–4. PubMed doi:10.1016/0140-6736(91)90207-6

Koelle K, Rodo X, Pascual M, Yunus M, Mostafa G. Refractory periods and climate forcing in cholera dynamics. Nature. 2005;436:696–700. PubMed doi:10.1038/nature03820

Previous Experiences with Mass OCV Campaigns in Complex Emergency Settings

Legros D, Paquet C, Perea W, Marty I, Mugisha NK, Royer H, et al. Mass vaccination with a two-dose oral cholera vaccine in a refugee camp. Bull World Health Organ. 1999;77:837–42. PubMed

World Health Organization. Use of the two-dose oral cholera vaccine in the context of a major natural disaster. Report of a mass vaccination campaign in Aceh Province, Indonesia, 2005. Geneva: World Health Organization; 2006.

Draft Background Paper on the Integration of Oral Cholera Vaccines into Global Cholera Control Programs. Presented to SAGE in October 2009. Geneva: WHO Strategic Advisory Group of Experts on Immunization; 2009.

Recent Published Models Regarding Reactive Cholera Vaccination in Epidemic Settings

Andrews JR, Basu S. Transmission dynamics and control of cholera in Haiti: an epidemic model. Lancet. 2011;377:1248–55. PubMed doi:10.1016/S0140-6736(11)60273-0

Chao DL, Halloran ME, Longini IM. Vaccination strategies for epidemic cholera in Haiti with implications for the developing world. Proc Natl Acad Sci U S A. 2011;108:7081–5. PubMed doi:10.1073/pnas.1102149108

Reyburn R, Deen JL, Grais RF, Bhattacharya SK, Sur D, Lopez AL, et al. The case for reactive mass oral cholera vaccinations. PLoS Negl Trop Dis. 2011;5:e952. PubMed doi:10.1371/journal.pntd.0000952

Cholera Vaccine Stockpile

Danovaro-Holliday MC. Ad-hoc scientific consultation on potential role of cholera vaccination in the Americas in the context of the 2010 outbreak in the Hispaniola island. Global Immunization News. Geneva: World Health Organization (WHO). 28 January 2011.

World Health Organization. Meeting of the Strategic Advisory Group of Experts on Immunization, October 2009 – conclusions and recommendations. Geneva: World Health Organization; 11 December 2009.

World Health Organization. Potential use of oral cholera vaccines in emergency situations. Report of a WHO meeting. Geneva: World Health Organization; May 1999.

Chao DL, Halloran ME, Longini IM. Vaccination strategies for epidemic cholera in Haiti with implications for the developing world. Proc Natl Acad Sci U S A. 2011;108:7081–5. PubMed doi:10.1073/pnas.1102149108

Waldor MK, Hotez PJ, Clemens JD. A national cholera vaccine stockpile — a new humanitarian and diplomatic resource. N Engl J Med. 2010;363:2279–82. PubMed doi:10.1056/NEJMp1012300

Applied Strategies Consulting. GAVI Vaccine Investment Strategy: Cholera Analysis: GAVI; 2008.

Cholera Outbreaks in Other Countries Coincident with the Outbreak in Haiti

ProMED-mail. Cholera, Diarrhea and Dysentry Update 2010 (26). ProMED-mail 1 Nov 2010. http://www.promedmail.org

ProMED-mail. Cholera, Diarrhea and Dysentry Update 2010 (27). ProMED-mail 9 Nov 2010. http://www.promedmail.org
