# Supplementary material for: Considerations for Oral Cholera Vaccine Use during Outbreak after Earthquake in Haiti, 2010−2011
Source: Emerg Infect Dis. 2011 Nov;17(11):2105–12. doi: 10.3201/eid1711.110822 (PMC3310586; doi:10.3201/eid1711.110822)
Supplement: Technical Appendix 2 — Real-Time Modeling of Estimated Cholera Vaccine Impact on the Cholera Outbreak in Haiti, Centers for Disease Control and Prevention, December 2010. [file 11-0822-Techapp2_5p.pdf]

Article DOI: http://dx.doi.org/10.3201/eid1711.11082211

Considerations for Oral Cholera Vaccine Use during 2010–2011 Outbreak after Earthquake in Haiti

Technical Appendix 2

Real-Time Modeling of Estimated Cholera Vaccine Impact on the Cholera Outbreak in Haiti, Centers for Disease Control and Prevention, December 2010

A modified susceptible-infected-removed model with an environmental (water) component was used during early stages of the Haiti cholera outbreak to make projections about future cholera cases and hospitalizations. The model incorporated separate epidemic curves of cholera hospitalizations for Haiti's 10 departments (geographic regions) and the Port-au-Prince metropolitan area with some spillover between departments. The model was calibrated by using surveillance data from the Haitian Ministère de la Santé Publique et de la Population (MSPP). Basic model parameters included 1) proportion of infected case-patients who are symptomatic = 39.6% (*1*); 2) proportion of symptomatic case-patients who are hospitalized = 40% (MSPP Surveillance Data, November 16, 2010); 3) proportion of symptomatic case-patients who die = 2.2% (MSPP surveillance data December 7, 2010, the end of the 7th week and the most recent date used for this model iteration); 4) mean length of illness = 5 days; and 5) mean survival of *Vibrio cholerae* spp. in water = 30 days. Other model parameters, such as the amount of exposure to contaminated water, infectivity of contaminated water, and rate of geographic spread of *Vibrio* in water, were varied to produce the smallest error between the modeled and reported number of weekly hospitalizations by department, and those parameters were used to project the future extent of the outbreak.

On December 16, 2010, this model was used in an exercise, which projected the impact of a program that would begin distributing vaccine in 1 month (starting January 16, 2011). The analysis was run by using R version 2.8.1 (R Foundation for Statistical Computing, Vienna, Austria).

The following additional assumptions regarding cholera vaccine use were made: 1) use of 2-dose Dukoral (Crucell, Stockholm, Sweden) vaccine regimen for all eligible persons; 2) vaccination rate of 10,000 doses per day; 3) 80% of first dose recipients would receive a second dose, given 2 weeks after the first dose; 4) single-dose vaccine efficacy of 50% at 2 weeks; 5) 2-dose vaccine efficacy of 85% from 8.5 days to 6 months, then declining to ≈62% at 1 year, 58% at 2 years, and 18% at 3 years according to an exponential decay regression curve fit; 6) 10% vaccine wastage; and 7) loss of immunity for recovering case-patients beginning at 6 months and progressing at the same rate as vaccinated persons.

Results: Estimated Impact of Cholera Vaccination on Numbers of Cholera Cases and Hospitalizations in Haiti

The CDC model (based on data as of December 16, 2010) predicted a total of 577,878 cholera cases, including 231,151 hospitalizations during a 1-year period in the absence of any vaccination campaign. Figure shows the estimated impact of vaccination scenarios on cholera hospitalizations; impact estimates for numbers of cases were parallel (Table). The administration of 300,000 vaccine doses (≈1.5% of Haiti's population; Haiti Institute of Statistics, 2009 population estimates, http://www.ihsi.ht/pdf/projection/POPTOTAL&MENAGDENS_ESTIM2009.pdf) in all Departments proportional to their population was projected to prevent an estimated 3.8% of all cases and hospitalizations in the first year of the outbreak; the proportional decrease in cases and hospitalizations would have been 9.2% with 1 million doses (≈5% of Haiti's population) and 11.8% with 3 million doses administered (≈15% of Haiti's population). Vaccination in Artibonite Department only would have had a lower impact compared to the strategy involving all 10 departments.

Limitations

The model had several limitations: 1) the model was based on the first 7 weeks of surveillance data, which may not have been consistently reported between locations or different time periods; 2) this model assumed that epidemic dynamics governing disease spread and treatment did not vary by department (other than by population density); 3) the model did not

assess epidemic dynamics at a subdepartmental level; 4) the model did not have a term for person-to-person spread through concepts such as hyperinfectious vibrios, which were included in other later models; 5) the model does not have a seasonal component; and 6) effects of health interventions (besides vaccination) were not considered.

References

1. Swerdlow DL, Mintz ED, Rodriguez M, Tejada E, Ocampo C, Espejo L, et al. Severe life-threatening cholera associated with blood group 0 in Peru: implications for the Latin American epidemic. J Infect Dis. 1994;170:468–72. PubMed doi:10.1093/infdis/170.2.468

2. Hartley DM, Morris JG Jr, Smith DL. Hyperinfectivity: a critical element in the ability of *V. cholerae* to cause epidemics? PLoS Med. 2006;3:e7. PubMed doi:10.1371/journal.pmed.0030007

3. Bertuzzo E, Azaele S, Gatto AMM, Rodriguez-Iturbe I, Rinaldo A. On the space-time evolution of a cholera epidemic. Water Resources Research. 2008;44.

Table. Projected numbers of cholera cases and hospitalizations in the first year, by vaccination scenario, Haiti, 2011

Vaccination scenario	Projected no. cases	Projected no. hospitalizations*
No vaccination	577,878	231,151
300,000 OCV* doses in Artibonite Department only	564,970	225,988
300,000 OCV doses in all departments proportional to population	555,808	222,323
1 million OCV doses in Artibonite Department only	559,253	223,701
1 million OCV doses in all departments proportional to population	524,900	209,960
3 million OCV doses in Artibonite Department only	558,393	223,357
3 million OCV doses in all departments proportional to population	509,753	203,901

*OCV, oral cholera vaccine.

Figure (see following pages). Projected impact of oral cholera vaccine (OVC) use on number of cholera hospitalizations, by vaccination strategy (no. of doses and geographic target), Haiti, 2011. A) Projected weekly hospitalizations in Haiti with use of 300,000 OCV doses (≈1.5% of Haiti’s population vaccinated); B) projected weekly hospitalizations in Haiti with use of 1 million OCV doses (≈5% of Haiti’s population vaccinated); and C) projected weekly hospitalizations in Haiti with use of 3 million OCV doses (≈15% of Haiti’s population vaccinated).

A

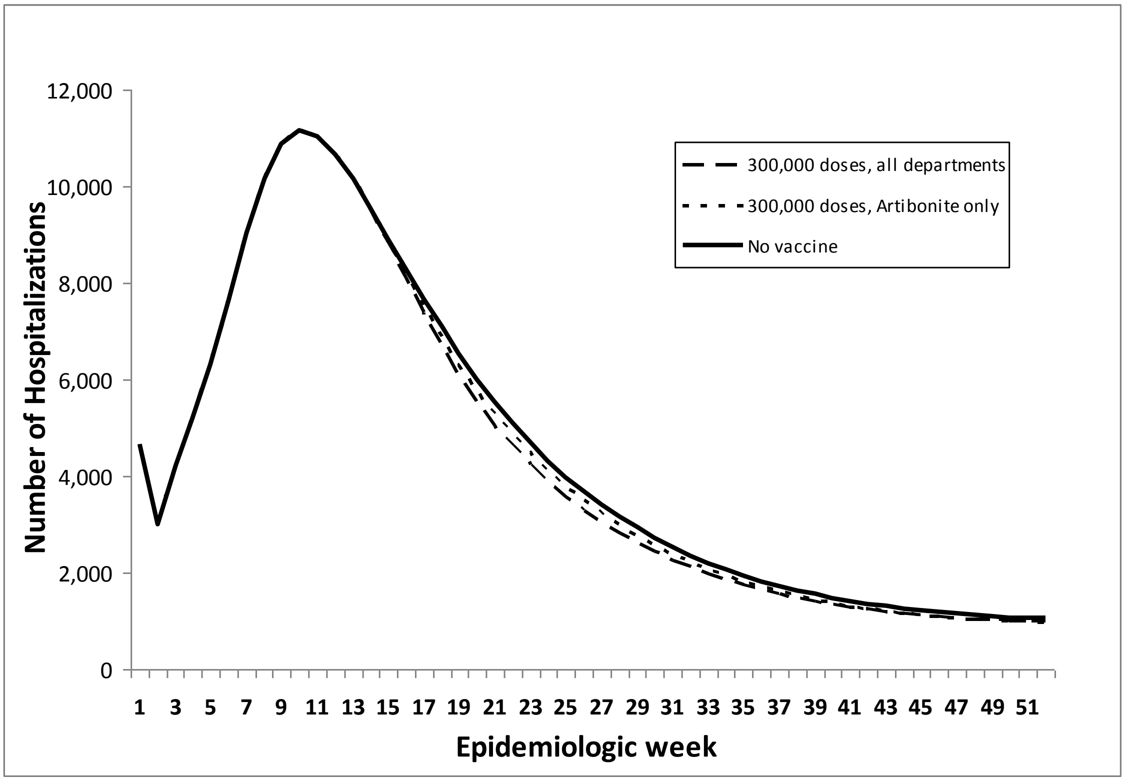
12,000
10,000
8,000
6,000
4,000
2,000
0
Number of Hospitalizations
1 3 5 7 9 11 13 15 17 19 21 23 25 27 29 31 33 35 37 39 41 43 45 47 49 51
Epidemiologic week
300,000 doses, all departments
300,000 doses, Artibonite only
No vaccine

B

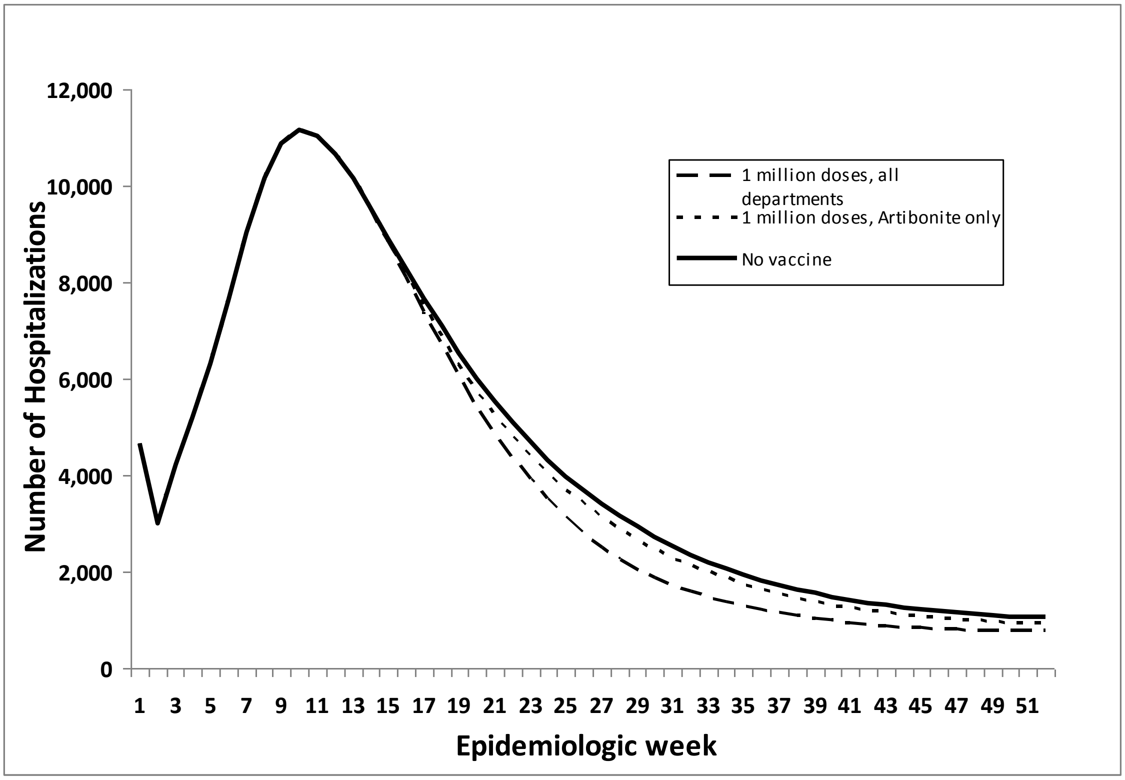
12,000
10,000
8,000
6,000
4,000
2,000
0
Number of Hospitalizations
1 3 5 7 9 11 13 15 17 19 21 23 25 27 29 31 33 35 37 39 41 43 45 47 49 51
Epidemiologic week
1 million doses, all departments
1 million doses, Artibonite only
No vaccine

c

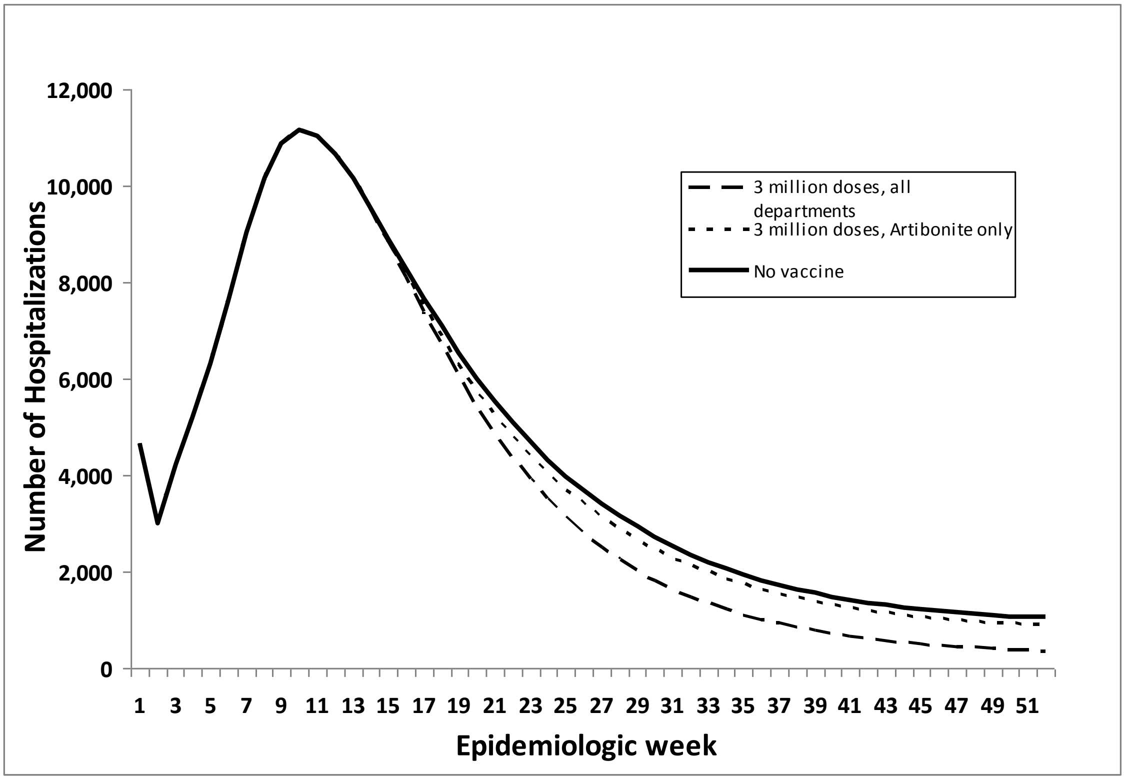

12,000
10,000
8,000
6,000
4,000
2,000
0
Number of Hospitalizations
1 3 5 7 9 11 13 15 17 19 21 23 25 27 29 31 33 35 37 39 41 43 45 47 49 51
Epidemiologic week
3 million doses, all departments
3 million doses, Artibonite only
No vaccine
